# Mutations in Genes Encoding Subunits of the RNA Exosome as a Potential Novel Cause of Thrombotic Microangiopathy

**DOI:** 10.3390/ijms25147604

**Published:** 2024-07-11

**Authors:** Kioa L. Wijnsma, Anne M. Schijvens, Romy N. Bouwmeester, Lonneke A. M. Aarts, Lambertus (Bert) P. van den Heuvel, Charlotte A. Haaxma, Nicole C. A. J. van de Kar

**Affiliations:** 1 Department of Pediatric Nephrology, Amalia Children’s Hospital, Radboud University Medical Center, 6525 GA Nijmegen, The Netherlandsromy.bouwmeester@radboudumc.nl (R.N.B.); bert.vandenheuvel@radboudumc.nl (L.P.v.d.H.); nicole.vandekar@radboudumc.nl (N.C.A.J.v.d.K.); 2 Department of Pediatrics, Amalia Children’s Hospital, Radboud University Medical Center, 6525 GA Nijmegen, The Netherlands; lonneke.aarts@radboudumc.nl; 3 Department of Pediatric Neurology, Amalia Children’s Hospital, Radboud University Medical Center, 6525 GA Nijmegen, The Netherlands; charlotte.haaxma@radboudumc.nl

**Keywords:** thrombotic microangiopathy, hemolytic uremic syndrome, RNA exosome

## Abstract

Thrombotic microangiopathy (TMA) in association with RNA exosome encoding mutations has only recently been recognized. Here, we present an infant (female) with an *EXOSC5* mutation (c.230_232del p.Glu77del) associated with the clinical phenotype known as CABAC syndrome (cerebellar ataxia, brain abnormalities, and cardiac conduction defects), including pontocerebellar hypoplasia, who developed renal TMA. At the age of four months, she presented with signs of septic illness, after which she developed TMA. A stool culture showed rotavirus as a potential trigger. The patient received eculizumab once, alongside supportive treatment, while awaiting diagnostic analysis of TMA, including genetic complement analysis, all of which were negative. Eculizumab was withdrawn and the patient’s TMA recovered quickly. A review of the literature identified an additional four patients (age < 1 year) who developed TMA after a viral trigger in the presence of mutations in *EXOSC3*. The recurrence of TMA in one of these patients with an *EXOSC3* mutation while on eculizumab treatment underscores the apparent lack of responsiveness to C5 inhibition. In conclusion, mutations in genes influencing the RNA exosome, like *EXOSC3* and *EXOSC5*, characterized by neurodevelopment and neurodegenerative disorders could potentially lead to TMA in the absence of complement dysregulation. Hence, these patients were likely non-responsive to eculizumab.

## 1. Introduction

Thrombotic microangiopathy (TMA) is a well-known concept within the field of nephrology, as it is used as an umbrella term to describe the triad of mechanical hemolytic anemia, thrombocytopenia, and ischemia in end organs, with acute kidney injury being the most prominent feature. Numerous causes of TMA have been reported, but in pediatric patients with TMA more than 95% of cases are related to one of the following three entities: (i) infection with Shiga toxin-producing Escherichia coli (STEC) leading to hemolytic uremic syndrome (HUS; STEC-HUS), (ii) infection with Streptococcus pneumoniae leading to HUS, or (iii) HUS as a result of complement dysregulation, referred to as atypical HUS (aHUS) [1]. However, every year new entities leading to TMA are described, often resulting from extensive genetic analysis revealing pathogenic mutations that predispose for the development of TMA.

Recently, the development of TMA in relation to RNA exosome encoding mutations has been described [2,3]. Little is known about the potential role of the RNA exosome in the development of TMA. The RNA exosome is a ubiquitously expressed complex composed of various exosome component (EXOSC) proteins. It has a critical function in the processing and degradation of both coding and non-coding RNA, and is critical for the assembly of the ribosome [4]. Alterations in ribosome production could ultimately lead to cell cycle arrest and apoptosis [4]. The RNA exosome complex comprises a total of ten subunits, of which six subunits (EXOSC4–9) form the core ring of the complex, while the first three (EXOSC1–3) form the cap of the exosome. Genetic mutations in these subunits have been described to alter the stability or conformations of proteins, potentially leading to decreased function of the exosome. Mutations in many of these structural subunits have been described in relation to neurodevelopmental and neurodegenerative diseases, often in the presence of pontocerebellar hypoplasia (PCH) [5]. A wide range of phenotypic severity is described within mutations in one subunit, while, on the other hand, a high rate of similarities in phenotype is reported between mutations in different subunits [5,6].

We present a patient with an *EXOSC5* mutation leading to the clinical phenotype currently known as CABAC syndrome (cerebellar ataxia, brain abnormalities, and cardiac conduction defects), including pontocerebellar hypoplasia, who also developed TMA. Subsequently, we discuss the few known cases in the literature of mutations in the RNA exosome described in relation to TMA [2,3,7,8].

## 2. Case Description

In our tertiary care center in the Netherlands, a patient with an *EXOSC5* mutation was diagnosed with TMA at the age of 4 months. The patient (F) was born small for her gestational age (<p 2.3) at 35 weeks of gestation after the first pregnancy of consanguineous parents of Syrian descent. The pregnancy was complicated by the suspicion of cerebellar hypoplasia, based on antenatal ultrasounds. The patient had several dysmorphic features: frontal bossing, bitemporal narrowing, down-slanted eyes, a prominent nose with a broad nasal tip, a prominent philtrum, low-set and posteriorly rotated ears, and full lips. Moreover, she had a small thorax and long fingers and thumbs. The cerebral MRI showed pontocerebellar hypoplasia, an immature gyral pattern, and a small aspect of the neurocranium with a small biparietal diameter. Repeated cardiac evaluations were performed because of previously described severe cardiac conduction problems, but showed no significant abnormalities [9]. Evaluation of the eyes indicated intermittent esotropia of the right eye, poor Bell’s reflex on both sides, but no ophthalmic abnormalities. An audiological examination at five weeks revealed mixed (conductive and sensorineural) hearing loss in both ears. Genetic diagnostics revealed a homozygous mutation in the *EXOSC5* gene (c.230_232del and p.Glu77del), which explained most, if not all, of the above-mentioned clinical features (within the spectrum of cerebellar ataxia, brain abnormalities, and cardiac conduction defects (CABAC) syndrome). During the first seven weeks of life, the patient was admitted to the hospital for feeding difficulties and respiratory problems (irregular breathing with central and obstructive apneas). Her feeding difficulties were likely due to a combination of neurological coordinative problems, axial hypotonia, mild respiratory distress, and her mucosal stasis due to facial dysmorphic features. Therefore, the girl was mostly fed via a nasogastric tube. At 12 weeks, a typical skin configuration with diffuse mottled discoloration classified as livedo reticularis was noticed during a regular follow-up appointment at the outpatient clinic. No relation with other symptoms could be identified, and the skin configuration remained present during the months thereafter. At the age of three months, there were no signs of psychomotor development, but instead, frequent dysphoria and increasing pyramidal and dystonic muscle tone had developed, as well as non-febrile seizures, including status epilepticus, for which various anti-epileptic drugs (levetiracetam, valproic acid, chloral hydrate, and midazolam) were administered.

### 2.1. Onset of TMA

**Figure 1:**
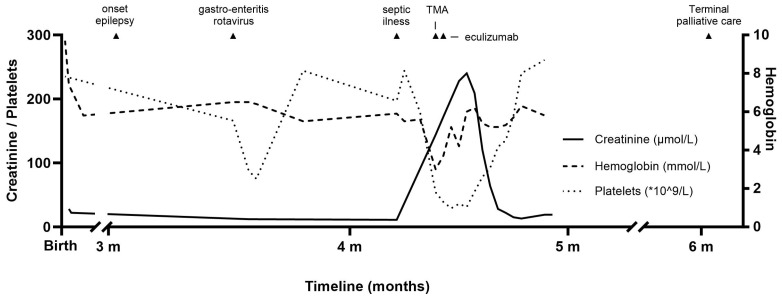
Timeline of laboratory values and clinical events. The horizontal axis shows the age of the patients. On the left *Y*-axis, creatinine and platelet levels are depicted; on the right Y-axis, hemoglobin is depicted. Small triangles represent clinical events.

Two weeks later, she was admitted with gastro-enteritis due to a rotavirus. Three weeks thereafter, at the age of four months, the patient presented at the emergency department with signs of septic illness. Rotavirus was still present in stool cultures. Due to septic presentation, the patient was admitted and started on ceftriaxone after blood cultures were drawn. Five days after presentation, peripheral edema, oliguria, and hypertension developed. Laboratory evaluation showed the triad of TMA with, among other things, LDH levels up to 2000 U/L, and undetectable haptoglobin and schistocytes in the peripheral blood smear (Figure 1). Bilirubin levels were low and the reticulocyte count was slightly elevated. A routine workup was performed to find the underlying cause of TMA in this patient. A fecal diagnostic was negative for STEC-infection, and no antibodies to LPS (lipopolysaccharide) of STEC were detected. No other infections were detected: blood cultures remained negative, an extensive respiratory panel remained negative (also for SARS-CoV-2), and CMV, EBV, and parvovirus serology were negative. Pneumococcal HUS was considered, but seemed unlikely given initial clinical improvement with no fever during admission, inflammation markers that remained low, and ceftriaxone use for five days already. In addition, Coombs was negative (false positive Coombs occurs in >60% of pneumococcal HUS) and anti-DNAse and AST were both below the detection limit. Diffuse intravascular coagulation (DIC) as a cause of TMA was considered unlikely, as hemoglobin levels, platelet counts, and LDH normalized without heparin treatment. The patient exhibited signs of TMA five days after the onset of sepsis and had been receiving antibiotics since admission, leading to an improvement in both fever and CRP levels. In addition, it has been reported that sepsis-associated DIC may reduce ADAMTS13 levels due to consumption resulting from high levels of von Willebrand factor multimers released by perturbed endothelium, whereas in this case ADAMTS13 levels were normal [10]. Additionally, there were no initial indications of DIC, as evidenced by normal kidney function, platelet counts, and the absence of hemolysis. PT values support this, as they were normal. Fibrinogen or D-dimer levels were not determined. Thrombotic thrombocytopenic purpura (TTP) was excluded because of the presence of normal ADAMTS13 activity (42). As a complement-mediated TMA could not be excluded in this acute phase, the national aHUS working group was consulted. One gift of eculizumab (300 mg) was given, and genetic investigation of the complement genes, as well as the *DGKƐ* gene, was initiated (see Table 1 and Supplementary File S1). Additionally, supporting therapy was started, including erythrocyte transfusions, fluid restriction, a protein- and electrolyte-restricted diet, and antihypertensive and diuretic drugs.

### 2.2. Genetic Analysis

On religious grounds, the parents waived amniocentesis or any further prenatal genetic diagnostics. Postpartum genetic analysis revealed a homozygous mutation in *EXOSC5*: Chr19(GRCh37):g.41898803_41898805del NM_020158.4:c.230_232del p.(Glu77del), OMIM #619576. This mutation is a frame deletion of one amino acid in ExoRNase_PNdom1 exoribonuclease, phosphorolytic domain 1 of the EXOSC5 protein. This variant had not yet been described in The Genome Aggregation Database (GnomAD; http://gnomad.broadinstitute.org). As there are few to no homozygous variants (incl. missense variants or in-frame deletions) observed in the coding parts of the EXOSC5 gene in the GnomAD, this variant was considered likely pathogenic. As previously described, the parents were consanguineous (of Syrian descent) and both heterozygous carriers of this *EXOSC5* variant. Of note, multiple homozygous areas (>400 Mb of autosomal genome) were seen within data of the index patient.

After the development of TMA, extensive additional genetic analysis was performed to examine genes associated with TMA (Table 1 and Supplementary File S1). No pathogenic mutation associated with the development of TMA was identified. The complement factor H (*CFH*) risk haplotype (homozygous) was detected, but considered not relevant in the absence of any other complement mutation associated with the development of TMA.

### 2.3. Ouctome

No complement abnormalities, such as autoantibodies against factor H or pathogenic mutations in complement genes, were detected. In our center, we have the capability to receive a genetic analysis in cases of aHUS within 14 days to support decision making regarding the need for eculizumab. The eculizumab serum trough level was checked after 14 days and remained the within the therapeutic range (142 ug/mL). However, the patient received only one dose of eculizumab in the absence of signs indicative of complement dysregulation. Due to the severity of the neurological disorder, with severe developmental delay, seizures, and very limited social interaction, a conservative approach was chosen. Creatinine levels, hemoglobin levels, platelet count, and LDH normalized within two weeks (Figure 1). As the *EXOSC5* mutation is not associated with complement regulation, the improvement in our patient was likely due to supporting therapy and elimination of the viral infection rather than to eculizumab therapy. However, more research is needed regarding the role of the RNA exosome in relation to TMA and its potential contribution to the complement system. In the weeks thereafter, the patient was readmitted twice with epileptic seizures, fever, and respiratory failure. No further blood samples (among other samples, to look for recurrence of TMA) were taken, as terminal palliative care was initiated. Finally, due to severe progressive neurological disorder and respiratory failure, the patient passed away at the age of 6 months.

## 3. Discussion

After an extensive literature search (see Supplementary File S2 for the extensive search strategy), we found four additional cases describing TMA in patients with mutations in the *EXCOSC3* gene. It is noteworthy that we found an additional article from 2003 by Rudnik-Schöneborn et al., which described a patient with PCH who developed HUS; however, no genetic data were presented [11]. In all four patients, the clinical course was preceded by a viral trigger (Table 1) [2,3]. Moreover, all patients received multiple doses of eculizumab, yet clinical improvement attributable to eculizumab was highly unlikely. Moreover, one patient experienced TMA recurrence while being treated with eculizumab. Here, we report a case of TMA in a child with homozygous mutations in the *EXOSC5* gene, which encodes another subunit in the RNA exosome.

*EXOSC5* and *EXOSC3* are present on the same face of the RNA exosome core complex. Disturbances in the interaction between these subunits could explain the overlapping clinical presentation, as thoroughly described for the neurological phenotype. Of the ten patients with an EXOSC5 mutation previously described in the literature, none showed kidney involvement [5,6,7]. However, an intact RNA exosome is essential for the assembly of the ribosome [4]. RNAseq experiments performed on human kidney tissue display approximately 10 EXOSC5 transcripts per million, and approximately 14 EXOSC5 transcripts per million renal endothelial cells. In the Human Protein Atlas for the kidney, the EXOSC5 protein is expressed in other locations in renal endothelial cells. The EXOSC5 protein is present in the nucleus and the cytoplasm, and is involved in (i) RNA processing and proper maturation of stable RNA species; (ii) degradation of histon RNA; (iii) ribonucleolysis; (iv) reacting upstream of, or within the defense response to, a virus; (v) general mRNA turnover, and specifically degradation of unstable mRNAs containing AU-rich elements (AREs) at 3′-UTR; and (vi) ribosome assembly. It is hypothesized that infection causes an accumulation of viral RNA or defective processing of (viral) RNA in the cell, inducing ribosomal dysfunction in the endothelial cells resulting in apoptosis, and consequently leading to the cascade resulting in TMA [2,3]. This phenomenon is already known from the most common cause of TMA in children, STEC-HUS. Here, intracellular Shiga toxin interferes with the ribosome and induces the inhibition of protein synthesis and cell death, which is most prominent on glomerular endothelial cells [12,13,14]. Notably, this effect is temporary in the case of STEC-HUS, but may be recurrent in cases of a genetic mutation. Interestingly, in multiple patients with PCH/CABAC based on a pathogenic mutation in subunit two of the tRNA splicing endonuclease complex (*TSEN2*), TMA has also been described [2,8]. This may suggest a potential role for the RNA pathway in endothelial dysfunction, which could ultimately lead to the development of TMA. However, at this moment we cannot rule out that the previously noted functions of *EXOSC5* may influence the expression of other genes or proteins involved in TMA, which could explain the development of TMA in some of these patients. More research is needed to unravel the role of mutations in the RNA exosome and the development of TMA.

## 4. Conclusions

In summary, TMA has now been reported in a total of five patients with a mutation in one of the *EXOSC* genes, which are associated with congenital neurological abnormalities followed by a neurodegenerative disease course. In this case report, we summarized these cases and consequently aimed to emphasize the importance of adding mutations in the *EXOSC* genes to the potential causes of TMA in infants. This is highly important for pediatricians to ensure early detection of TMA in these patients and subsequent initiation of optimal supportive treatment. Importantly, in patients with mutations in *EXOSC3/EXOSC5*, TMA can occur that appears not to be not linked to complement dysregulations; therefore, these patients are unlikely to benefit from eculizumab therapy. Future studies must unravel the role of the dysfunctional RNA exosome in the pathophysiology of TMA.

## Figures and Tables

**Table 1:** Patients with a mutation in one of the *EXOSC* genes with TMA.

	Patient 1 [3]	Patient 2 [3]	Patient 3 [2]	Patient 4 [2]	Patient 5 Index (This Report)
**Gender**	M	M	F	M	F
**Age at onset of TMA symptoms**	4 months	4.5 months	8 months	7 months	4 months
**Gestational age**	26 weeks	—	term	term	35 + 2 weeks
**Abnormalities since birth**	Microcephaly, joint contractures, axial hypotonia	—	Pontocerebellar hypoplasia	Pontocerebellar hypoplasia	Cerebellar hypoplasia, symmetrical fetal growth restriction
**Presenting symptoms**	Fever, mild respiratory symptoms	Pyrexia, diarrhea, reduced drinking	Bronchiolitis in need of mechanical ventilation	Lower respiratory tract infection in need of mechanical ventilation	Fever, illness
**(Potential) trigger**	SARS-CoV-2 infection	SARS-CoV-2 infection	RSV infection	Unspecified respiratory virus	Rotavirus infection
**Timing of TMA symptoms**	5 days after onset of COVID-19	5 days after onset of COVID-19	At presentation	At presentation	6 days after presentation with fever
**Complement analysis**					
**CFH**	Normal	Normal	Slightly elevated	Normal	ND
**CFI**	Normal	Normal	Slightly decreased	Normal	ND
**CFB**	Normal	Normal	ND	ND	ND
**CFD**	Normal	Normal	ND	ND	ND
**Complement activity (CH50)**	Normal	Normal	—	—	Normal
**Anti-CFH autoantibodies**	No antibodies	No antibodies	No antibodies	No antibodies	No antibodies
**sC5b-9**	456 ng/mL Slightly above normal range	311 ng/mL Slightly above normal range	ND	ND	ND
**Antibodies against LPS O26, O55, O103, O157**	—	—	—	—	No antibodies
**Fecal diagnostics: STEC PCR**	Negative	Negative	Negative	Negative	Negative
**Genetic analysis for TMA**	No pathogenic mutation in *ADAMTS13*, *C3*, *CD46*, *CFB*, *CFH*, *CFHR1*, *CFHR2*, *CFHR3*, *CFHR4*, *CFHR5*, *CFI*, *DGKƐ*, *MMACHC*, *THBD*	No pathogenic mutation in *ADAMTS13*, *C3*, *CD46*, *CFB*, *CFH*, *CFHR1*, *CFHR2*, *CFHR3*, *CFHR4*, *CFHR5*, *CFI*, *DGKƐ*, *MMACHC*, *THBD*	No pathogenic mutation in *CFH*, *CFI*, *CD46*, *C3*, *CFB*, *DGKƐ*, *THBD*, *MTR*, *VTN*, *MMACHC*	No pathogenic mutation in *CFH*, *CFI*, *CD46*, *C3*, *CFB*, *DGKƐ*, *THBD*, *MTR*, *VTN*, *MMACHC*	No pathogenic mutation in *CFH* (including *MLPA*), *CFI*, *CD46*, *C3*, *CFB*, *DGKƐ*
**Gene associated with PCH**	*EXOSC3*	*EXOSC3*	*EXOSC3*	*EXOSC3*	*EXOSC5*
**Nucleotide alterations**	c.92G>C	c.92G>C	c.395A>C, c.341_343del	c.395A>C, c.92G>C	c.230_232del
**Consequence at protein level**	p.(Gly31Ala)	p.(Gly31Ala)	p.(Asp132Ala), (p.Glu114_Pro115delinsAla)	p.(Asp132Ala), p.(Gly31Ala)	p.(Glu77del)
**Zygosity**	Homozygous	Homozygous	Compound heterozygous	Compound heterozygous	Homozygous
**Eculizumab treatment**	Yes, 300 mg 0, 8, and 28 days after onset of TMA	Yes, 300 mg 0, 7, and 26 days after onset of TMA	Yes, single dose	Yes, continuous eculizumab for months	Yes, single dose, 300 mg
**Response to eculizumab**	Unresponsive	Unresponsive	No response	Initial improvement, TMA recurrence under eculizumab treatment after 4 months	Creatinine levels, hemoglobin levels, thrombocytes, and LDH were normalized within 2 weeks, likely due to conservative treatment rather than to eculizumab therapy
**Outcome**	Slight decrease in kidney function, persisting hypertension, persisting proteinuria.	Persisting hypertension, mild persisting proteinuria, normal kidney function.	Due to the underlying severity of the neurological disorder, palliative care was initiated.	Initially improvement was seen, relapse of TMA 4 months later with ongoing eculizumab treatment, patient died shortly thereafter.	Improvement of kidney function. Patient died at age 6 months after palliative care was initiated due to severity of the neurological disorder.

Abbreviations: ADAMTS13, a disintegrin and metalloprotease with thrombospondin motifs; AKI indicates acute kidney injury; Membrane cofactor protein, CD46; CFB, complement factor B; CFH, complement factor H; CFHR, complement factor H related; CFI, complement factor I; DGKƐ, Diacylglycerol kinase ɛ; MMACHC, methylmalonic aciduria and homocystinuria type C protein; MTR, 5-methyltetrahydrofolate-homocysteine methyltransferase; ND, not determined; PCH, pontocerebellar hypoplasia; THBD, thrombomodulin; TMA, thrombotic microangiopathy; VTN, vitronectin; —, not available in previously reported cases. For EXOSC5, transcript NM_020158.4 was used.

## Data Availability

Data are contained within the article.

## Supplementary Materials

The following supporting information can be downloaded at: https://www.mdpi.com/article/10.3390/ijms25147604/s1.
