# Supplementary material for: Mutations in Genes Encoding Subunits of the RNA Exosome as a Potential Novel Cause of Thrombotic Microangiopathy
Source: Int J Mol Sci. 2024 Jul 11;25(14):7604. doi: 10.3390/ijms25147604 (PMC11277116; doi:10.3390/ijms25147604)
Supplement: Supplementary file 1 [file ijms-25-07604-s001.zip › ijms-3055674-supplementary.pdf]

Supplement files

Supplement file 1

Extensive overview of genes tested after presentation with TMA:

- Complement Factor I (NM_000204.5)
- CD46 (MCP; NM_002389.4 and NM_172359.2)
- Complement Factor B (NM_001710.6)
- C3 (NM_000064.4)
- DGKE (NM_0003647.2)
- Complement Factor H (NM_000186.3)
- Complement factor H risk haplotype c.-331C>T (rs3753394), c.2016A>G (rs3753396) en c.2808G>T (rs1065489) (PMID: 14583443)
- MCP risk haplotype GGAAC c.-652G (rs2796267), c.-366G (rs2796268), c.989-78A (rs1962149), c.1127+638A (rs859705) en c.*897C (rs7144)
- MLPA ((Multiplex Ligation-dependent Probe Amplification, P236 CFH Region of MRC Holland) complement factor H combined with 20 home made probes to cover the CFH operon properly and screen for deletions and/or duplations in factor H operon on these positions.

Supplement file 2

For the literature search we used the following search terms in Pubmed:

"Exosome Multienzyme Ribonuclease Complex"[Mesh] OR "EXOSC9 protein, human" [Supplementary Concept] OR "EXOSC3 protein, human" [Supplementary Concept] OR "EXOSC7 protein, human" [Supplementary Concept] OR "EXOSC1 protein, human" [Supplementary Concept] OR "EXOSC2 protein, human" [Supplementary Concept] OR "EXOSC 10 protein, mouse" [Supplementary Concept] OR "EXOSC5 protein, human" [Supplementary Concept] OR "EXOSC8 protein, human" [Supplementary Concept] OR "EXOSC4 protein, human" [Supplementary Concept] OR "EXOSC10 protein, human" [Supplementary Concept] OR "DIS3 protein, human" [Supplementary Concept] OR “EXOSC1*”[tiab] OR “EXOSC2*”[tiab] OR “EXOSC3*”[tiab] OR “EXOSC4*”[tiab] OR “EXOSC5*”[tiab] OR “EXOSC6*”[tiab] OR “EXOSC7*”[tiab] OR “EXOSC8*”[tiab] OR EXOSC9*[tiab] OR "Exosome Complex*"[tiab] OR "Exosome Component*"[tiab] OR “RNA Exosome”[tiab] OR "Pontocerebellar Hypoplasia Type 1" [Supplementary Concept] OR “pontocerebellar hypoplasia*”[tiab] AND "Thrombotic Microangiopathies"[Mesh] OR” Hemolytic Uremic Syndrome*”[tiab] OR “HUS”[tiab] OR “HUSs”[tiab] OR “aHUS”[tiab] OR "Thrombotic Microangiopath*"[tiab] OR “Thrombocytopenia*”[tiab] OR “Thrombopenia*”[tiab]

We received 3 hits of which 2 describe *EXOSC* mutations and aHUS an one article discusses *TSEN2* mutation and aHUS.
